# Effects of high ambient temperature on ambulance dispatches in different age groups in Fukuoka, Japan

**DOI:** 10.1080/16549716.2018.1437882

**Published:** 2018-02-23

**Authors:** Kazuya Kotani, Kayo Ueda, Xerxes Seposo, Shusuke Yasukochi, Hiroko Matsumoto, Masaji Ono, Akiko Honda, Hirohisa Takano

**Affiliations:** ^a^ Department of Environmental Engineering, Kyoto University, Kyoto, Japan; ^b^ Environmental Science Section, Fukuoka City Institute of Health and Environment, Fukuoka, Japan; ^c^ Center for Health and Environmental Risk Research, National Institute for Environmental Studies, Tsukuba, Japan

**Keywords:** ambulance dispatch, temperature, distributed lag nonlinear model, age category, acute illness

## Abstract

**Background**: The elderly population has been the primary target of intervention to prevent heat-related illnesses. According to the literature, the highest risks have been observed among the elderly in the temperature–mortality relationship. However, findings regarding the temperature–morbidity relationship are inconsistent.

**Objectives**: This study aimed to examine the association of temperature with ambulance dispatches due to acute illnesses, stratified by age group. Specifically, we explored the optimum temperature, at which the relative health risks were found to be the lowest, and quantified the health risk associated with higher temperatures among different age groups.

**Methods**: We used the data for ambulance dispatches in Fukuoka, Japan, during May and September from 2005 to 2012. The data were grouped according to age in 20-year increments. We explored the pattern of the association of ambulance dispatches with temperature using a smoothing spline curve to identify the optimum temperature for each age group. Then, we applied a distributed lag nonlinear model to estimate the risks of the 85th–95th percentile temperature relative to the overall optimum temperature, for each age group.

**Results**: The relative risk of ambulance dispatches at the 85th and 95th percentile temperature for all ages was 1.08 [95% confidence interval (CI): 1.05, 1.12] and 1.12 (95% CI: 1.08, 1.16), respectively. In comparison, among age groups, the optimum temperature was observed as 25.0°C, 23.2°C, and 25.3°C for those aged 0–19, 60–79, and ≥80, respectively. The optimum temperature could not be determined for those aged 20–39 and 40–59. The relative risks of high temperature tended to be higher for those aged 20–39 and 40–59 than those for other age groups.

**Conclusions**: We did not find any definite difference in the effect of high temperature on ambulance dispatches for different age groups. However, more measures should be taken for younger and middle-aged people to avoid heat-related illnesses.

## Background

Owing to climate change, the frequency and intensity of hot weather have increased [1]. These changes in temperature are expected to increase heat-related health issues. Concurrently with the increase in extreme weather events owing to climate change, the proportion of the world’s population over 60 years of age is predicted to nearly double (12–22%) between 2015 and 2050 [2]. Japan leads the aging countries globally with 27% of the population older than 65 years [3]. There is a concern that the higher elderly population may amplify the heat-related health impact because of declines in physiological functions with age, particularly in terms of heat tolerance [4,5]. Taking this into consideration, the elderly has been the primary target population for intervention to prevent heat-related illnesses in Japan [6,7]. However, the impact of heat-related illnesses among various age groups has not yet been sufficiently clarified.

Several epidemiological studies have demonstrated that high temperature increases the risks for both mortality [8,9] and morbidity [10]. From the perspective of public health, it is relevant to identify vulnerable populations and to take effective precautionary measures to prevent heat-related illnesses. Age is an important factor that modifies the association between temperature and health. Several studies exploring the effect modifiers for the temperature–mortality relationship found that the elderly were susceptible to extreme temperatures [11–15]. However, there are relatively few studies characterizing the subgroups vulnerable to heat-related morbidity, and these results are inconsistent. While some studies examining the association of temperature with emergency department visits, hospitalizations, or ambulance dispatches imply that younger or middle-aged adults have higher heat-related risks [16–18], others claim that elderly people have higher heat-related risks during heat waves [19,20] and warm seasons [21,22].

A number of studies have examined the association of high temperatures with ambulance dispatches as a proxy for acute health outcomes [22–25]. Cases for ambulance dispatches include a wide range of nonfatal acute health outcomes compared with hospital-based data [22]. In Japan’s ambulance dispatch system, the citizen calls the telephone number 119 when they need emergency ambulance transport to the hospital. All case information is recorded and aggregated for each area, and has been utilized as a useful source of morbidity. For example, a study using ambulance dispatch data to investigate heatstroke characterized the temperature above which the risks started increasing and quantified the risks per unit increment in temperature [23].

Previous studies found a U-, V-, or J-shaped pattern of temperature–mortality relationships [25–28] with an optimum temperature at which the relative risks were lowest [29]. Studies using ambulance dispatches as health outcomes have also reported a similar pattern [11–14,24]. Susceptibility to high temperatures can be characterized by the optimum temperature level above which the health risk associated with temperature increases and the magnitude of the health risk associated with exceeding the optimum temperature. We hypothesized that the elderly may have a lower optimum temperature and a greater excess risk associated with high temperatures than younger people.

In this study, we examined the association between temperature and ambulance dispatches due to acute illnesses, stratified by age group. Specifically, we explored the optimum temperature level and quantified the risk associated with high temperature.

## Methods

### Location

The present study was conducted in Fukuoka City, which is located in the northern part of Kyushu Island, Japan. The city covers 341.3 km^2^, and its population was 1.46 million in 2010 according to the Japanese census. It has a mild, humid subtropical climate.

### Health outcome data

We obtained the daily ambulance dispatch data from 2005 to 2012 collected from all ﬁre stations within the city from Fukuoka City Office and the Ministry of Internal Affairs and Communications. In Japan, local governmental ﬁre defense headquarters provide prehospital emergency medical services as a public service. Anyone can use an ambulance free of charge by making a phone call to 119 [30]. After receiving the call, trained ambulance crews are dispatched, providing prehospital treatment and transporting the patients to the hospital. Each case record includes information regarding the patient’s age and sex, the cause of the dispatch, date and time of the dispatch, medical condition, and initial diagnosis. The medical condition and the initial diagnosis of those transported to the hospital were determined by an emergency medical doctor upon their arrival at the hospital. The cause of the dispatch is coded according to the International Statistical Classification of Diseases and Related Health Problems, 10th revision (ICD-10). A previous study [23] focused on the heatstroke-related ambulance dispatches to characterize the health impact of high temperature. We used all cases except for those related to injuries, pregnancy, and medical examinations for our analysis because high temperature not only increases the risk of heatstroke but also may exacerbate medical conditions, such as cardiovascular and respiratory diseases. Five categories were used to describe the patient’s medical condition in the data: mild, moderate, severe, dead, and other.

Ethical approval for this study was provided by the Ethics Committee of the Graduate School of Engineering at Kyoto University.

### Environmental data

Meteorological data regarding daily mean ambient temperature and relative humidity in Fukuoka city were provided by the Japan Meteorological Agency. A 24-hour mean ambient temperature and relative humidity values were calculated using hourly measurements. Data on air pollutants were also collected in Fukuoka City from the Ministry of the Environment and Fukuoka City Office.

### Statistical analyses

To examine the association of high temperatures and ambulance dispatch, we restricted the analyses to the warm season, from May to September. We divided the ambulance dispatch data into five age strata of 0–19 years, 20–39 years, 40–59 years, 60–79 years, and ≥80 years to examine the association between daily mean temperature and daily number of ambulance dispatches by each age category. We applied the following analyses both for all ages and for each age category.

First, we explored the pattern of the association of ambulance dispatches with daily mean temperature using a smoothing spline curve with 4 degrees of freedom (df) without confounders to identify the optimum temperature for each age group. If the pattern was U-, V-, or J-shaped, we selected the optimum temperature at which the relative risk associated with temperature was the lowest [29].

Second, we employed a quasi-Poisson regression using the distributed lag nonlinear model (DLNM) [26,27,31] to examine the association of high temperature with the daily number of ambulance dispatches. We used a cross-basis function of a natural cubic spline with 4 df [32] for temperature and 4 df for lag [25], with a maximum lagged effect up to 7 days based on previous studies [25,33]. We also included a natural cubic spline for date with 5 df per year to adjust for seasonality and long-term trends [32,34] and a natural cubic spline with 4 df for relative humidity [35–37] and particulate matter with an aerodynamic diameter of less than 2.5 µm (PM2.5) in the model [35,36,38,39] to control for potential confounders. We also adjusted for weekdays as the categorical variable [40] and weekends and holidays as the indicator variables [23,41,42]. Initially, we set the reference temperature at the optimum reference temperature observed in the risk curve for all ages to secure the comparability among the age groups. Since there is no gold standard for defining the extent of temperature effects in a specific temperature range, in this study we arbitrarily classified the temperature range into two, namely moderate (optimum reference temperature to 85th temperature percentile) and extreme high temperatures (optimum reference temperature to 95th temperature percentile). We further applied these two definitions to the rest of the age groups, to compare the relative risks per temperature range definition with respect to the age group. Results were presented as relative risks with 95% confidence intervals (CI). All analyses were performed in R 3.3.0 using the package DLNM.

## Results

From a total of 173,435 ambulance dispatches during the study period, 107,041 (61.7%), 22,003 (12.7%), and 20,049 (11.6%) cases were for acute illnesses, injuries, and traffic accidents, respectively. We extracted the data associated with ambulance dispatch due to acute illnesses and used these data for our analysis. The mean incident rate during the study period, calculated from the population size recorded in the 2010 Japanese census, was 1.9 cases per 1000 people/month and was highest for people aged ≥80 (7.7 per 1000 people/month) among all age categories. The daily number of ambulance dispatches due to acute illnesses ranged from 55 to 137 for all ages with a mean of 87.5 (Table 1) and larger on the weekend/holidays compared with that on the weekday.

**Table 1.** Summary of ambulance dispatch for acute illnesses by age group in Fukuoka city from May to September, 2005–2012.

			Daily number	
Age group	Total number	Incident rate (per 10^3^ person/month)^a^	Mean	Standard deviation
**All ages**	**107,041**	**1.9**	**87.5**	**14.2**
0–19	9863	0.9	8.1	3.2
20–39	22,329	1.3	18.3	4.7
40–59	21,655	1.4	17.7	4.7
60–79	32,633	2.8	26.7	6.4
≥80	20,561	7.7	16.8	5.7

^a^Incident rate was calculated based on the Japanese census in 2010.

Descriptive statistics on meteorological and air pollution data during the study period are shown in Table 2. The 85th and 95th percentiles of mean temperature during the study period were 29.4°C and 30.4°C, respectively.

**Table 2.** Descriptive statistics on daily meteorological and air pollution data in Fukuoka city from May to September, 2005–2012.

	Mean	Standard deviation	Minimum	25th percentile	Median	75th percentile	Maximum
Ambient temperature (°C)	24.9	3.9	13.1	21.9	25.3	28.4	31.8
Relative humidity (%)	70.4	10.0	36.6	64.0	70.3	77.3	95.8
PM2.5 (μg/m^3^)	19.5	11.5	2.9	11.1	16.6	25.0	94.8

In the scatter plots for all ages and each age category (Figure S-1), the predicted curves were U-shaped or hockey-stick-shaped. Optimum temperatures were observed as 23.5°C (37th percentile), 25.0°C (48th percentile), 23.2°C (35th percentile), and 25.3°C (50th percentile), for all ages, 0–19 years, 60–79 years, and ≥80 years, respectively (Table 3). For 20–39 and 40–59 years, we could not determine the optimum temperature because the predicted curve was not U-shaped during the warm season.

**Table 3.** Optimum temperature and its percentile for each age group.

Age group	Optimum temperature (°C)	Percentile of optimum temperature
**All**	**23.5**	**37**
0–19	25.0	48
20–39	–^a^	–^a^
40–59	–^a^	–^a^
60–79	23.2	35
≥80	25.3	50

^a^ The pattern was not U-shaped, and the optimum temperature was not identified.

**Figure 1.**
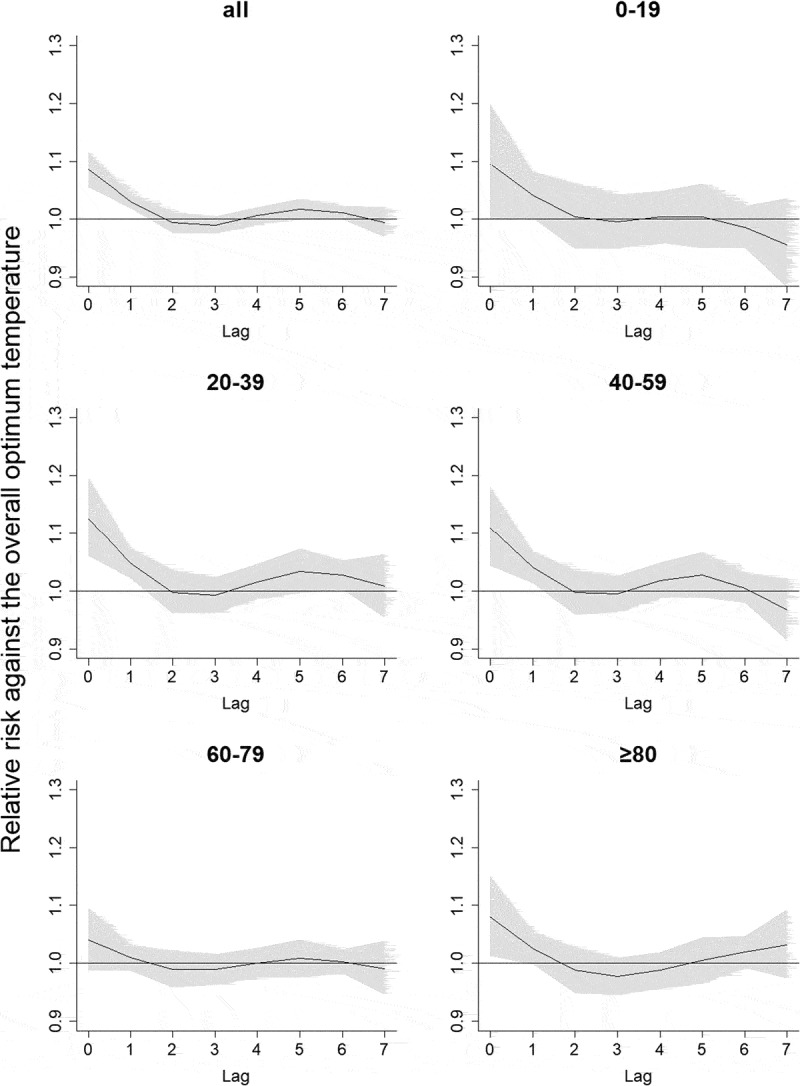
Lag patterns of the model at 85th percentile temperatures for all ages and each age category.

**Figure 2.**
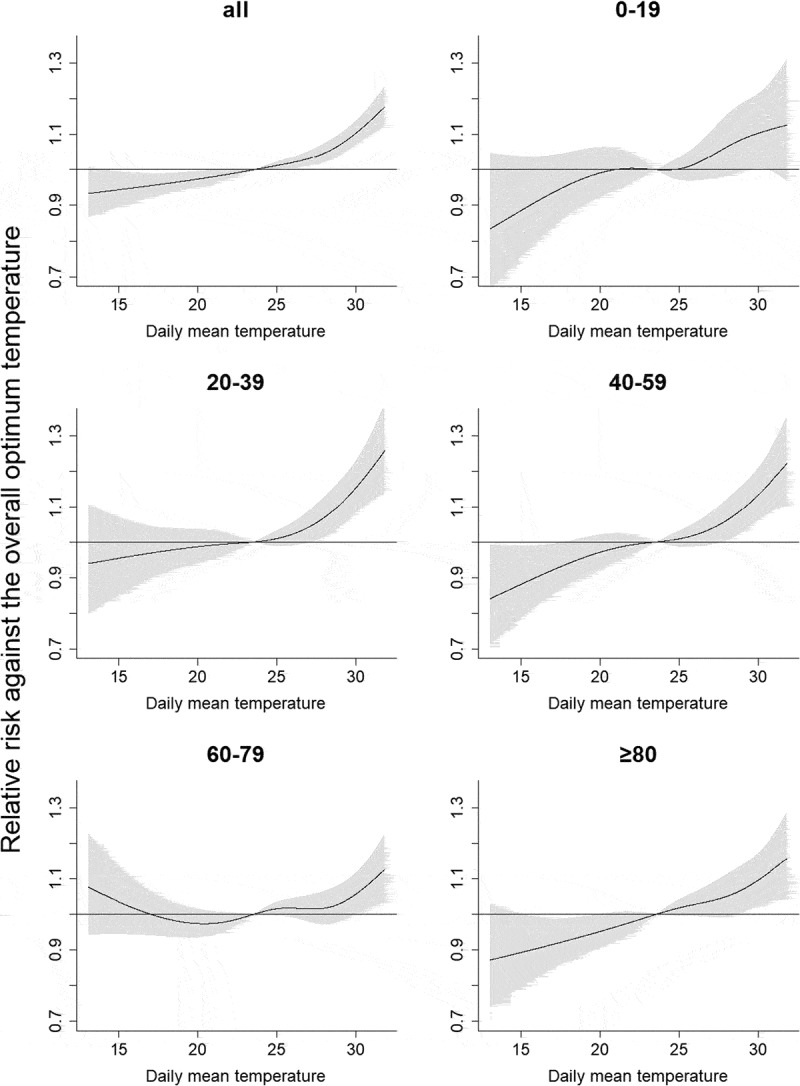
Relative risks against the overall optimum temperature at the same day for all ages and each age category.

Next, we applied DLNM to characterize the risks associated with high temperature (i.e., temperatures exceeding a reference temperature) relative to the overall optimum temperature, which refers to the optimum temperature for all ages (23.5°C). Figure 1 shows the lag patterns of risks of ambulance dispatches at the 85th percentile temperature relative to the reference temperature for each age category. The highest increase in the risk of ambulance dispatches was observed on the same day, and the lagged effect disappeared rapidly. Similar patterns were observed at the 95th percentile temperature. Figure 2 shows the temperature–risk relationship at the same day for all ages and each age category. We observed larger relative risks for those aged 20–39 and 40–59 years than other age groups.

**Figure 3.**
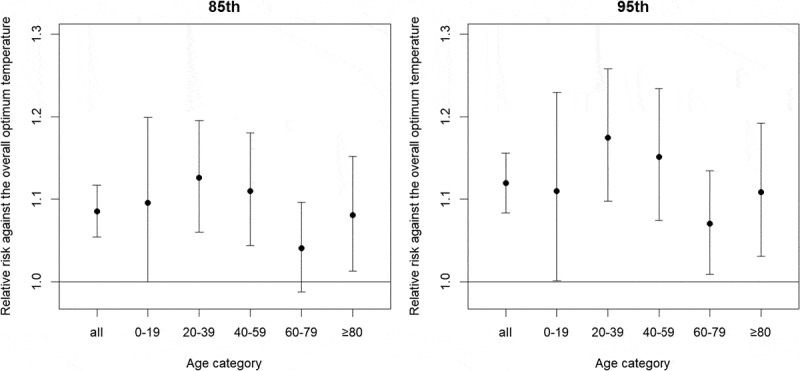
Relative risks of ambulance dispatches for each age category at the 85th (left) and 95th (right) percentile relative to the overall optimum temperature.

The relative risks of ambulance dispatches of lag 0 at the 85th and the 95th percentile temperature for all ages were 1.08 (95% CI: 1.05, 1.12) and 1.12 (95% CI: 1.08, 1.16), respectively (Figure 3). In comparison, among different age categories, the relative risk for 20–39 years tended to be highest, followed by 40–59 years, 0–19 years, and ≥80 years. The relative risk for 60–79 years tended to be the lowest. The shape of DLNM did not change substantially with or without each confounder.

In the analysis stratified by sex, we observed no clear difference in the pattern of temperature–risk relationship (Figure S-2), the optimum temperature (Table S-2), or the relative risks (Figure S-3) between men and women.

## Discussion

In this study, we explored the optimum temperature above which the health risk associated with temperature increases among different age categories using ambulance dispatch data. We also estimated the risk of ambulance dispatches at a high temperature and compared the risks among age categories. Although we hypothesized that the elderly have lower optimum temperatures and higher heat-related risks than younger people, our results did not confirm this assumption. No definite difference in optimum temperature was observed among age groups. Moreover, the risks of ambulance dispatches due to high temperature tended to be higher for the 20–39 and 40–59 age groups than for the elderly.

Although the biological mechanisms underlying the adverse health effects of heat have not been fully understood, experimental studies have shown that heat exposure increases platelets, red cell counts, blood viscosity, and plasma cholesterol levels [43], which may cause arterial thrombosis and result in cardiovascular diseases. Especially, a decline in physiological functions with age may decrease heat tolerance and render the elderly more susceptible to high temperature [4,5]. It has been reported that the elderly have poor thermoregulation, particularly due to less sweating and a weak cutaneous vascular response [44,45]. Another experiment has shown that older adults undergo greater thermal strain than younger adults during exposure to high temperatures [46].

The discrepancy between our hypothesis and the results of this study may be explained by the difference in behavioral patterns, in particular the actual duration of exposure to outdoor temperatures. It has been suggested that younger people may be more susceptible to high temperature than older people because of a longer working day [47]. Indeed, based on the 2006 Survey on Time Use and Leisure Activities by the Japan Ministry of Internal Affairs and Communications Statistics Bureau, the mean time spent working and commuting is much longer for younger people than for the elderly. Differences in leisure time duration and activities may further contribute to the higher risk of heat-related illness in the younger population. Besides, younger people spend more time on intense activities such as marathons and jogging than the elderly; this could increase their heat load [48]. Younger people may be engaged in physically demanding or outdoor work, for example in agriculture and construction sector, making them more vulnerable to high temperatures [49]. The previous studies also reported that high temperature was associated with a higher risk of work-related injuries [50,51] although the health impact of high temperature has not been fully studied in occupational settings.

The severity of the medical condition may affect our results. According to the distributions of medical conditions from ambulance dispatch data (Table S-1), mild and moderate cases accounted for most of the ambulance dispatches for younger people, while there were more serious conditions for the elderly. Mortality is an extreme health outcome and accounts for only a small percentage of all possible health outcomes. Mild health outcomes may be differently modified by age.

The results of this study have important implications for understanding the influence of age regarding the vulnerability of exposure to a high temperature. Although the elderly are more vulnerable to heat-related mortality than younger people, the present study suggests that younger people have a greater risk for mild to moderate heat-related illness, some of which need ambulance calls. There is a growing interest in interventions preventing heat-related illnesses, such as educational campaigns raising awareness of heat-related illnesses. Many of these campaigns put more emphasis on the elderly. However, we should be aware that younger and middle-aged people potentially have a high risk for heat-related illnesses as well as the elderly.

Our results imply the need to categorize age more precisely. The Organization for Economic Co-operation and Development defines ages 15–64 as the working-age population and ages 65 and over as elderly people [52]. Many epidemiological studies followed the same or similar age categorization. However, the mean life expectancy has been extended worldwide, and applying this age category definition to compare differences among age groups is not always appropriate, especially in developed counties with a longer life expectancy. For example, some gerontology studies divided elderly people into two age categories, young-old people and old-old people, and showed differences regarding health effects [53,54]. In our study, the risks of 60–79 years and ≥80 years tended to be different. With finer age categories, we could characterize the health risks more accurately.

A few limitations of this study should be noted. First, this is a single-city study. Therefore, it is possible that different patterns of effect modification by age will be observed in other areas where residents have different behavioral patterns. To generalize the results to all populations, it would be necessary to replicate the analysis in multiple areas. Second, we stratified age into ranges of 20 years. In order to characterize the vulnerability of children to extreme temperatures, it would be necessary to subdivide the data set into smaller age group, because children’s behavioral patterns and development vary greatly according to their age. Third, we applied the analysis to all-cause illnesses, not to cause-specific diseases due to the limited sample size. Further studies are needed to focus on the type of disease. Lastly, we used the ambulance dispatch data as a proxy of health outcomes. The ambulance data may not be available in some countries. It is also possible that some people visit emergency rooms by themselves, even if they had serious symptoms, and this case cannot be reflected in ambulance dispatch data.

## Conclusions

We found that the risk of ambulance dispatches increased as the temperature increases above the overall optimum temperature. The optimum temperature for all ages was 23.5°C and did not show a definite variance among age categories. The risks of ambulance dispatches above the optimum temperature tended to be higher for people in younger and middle-aged categories, although there was no statistically significant difference among different age categories. Therefore, the impact on health of high temperature should not be underestimated for young and middle-aged people as well as the elderly.

## Supplementary Material

Supplementary material

## Paper context

Several studies explored the temperature–mortality relationship and found that the elderly were susceptible to high temperature. However, there are relatively few studies characterizing the subgroups vulnerable to heat-related morbidity, and these results are inconsistent. The relative health risks of high temperature for younger and middle-aged people tended to be higher than that for the elderly people. More measures need to be taken for younger and middle-aged people to avoid heat-related illnesses.
