# Supplementary material for: Effects of high ambient temperature on ambulance dispatches in different age groups in Fukuoka, Japan
Source: Glob Health Action. 2018 Feb 23;11(1):1437882. doi: 10.1080/16549716.2018.1437882 (PMC5827789; doi:10.1080/16549716.2018.1437882)
Supplement: Supplementary material [file ZGHA_A_1437882_SM7165.zip › 180126_kotani_cle_SuppFigure.docx]

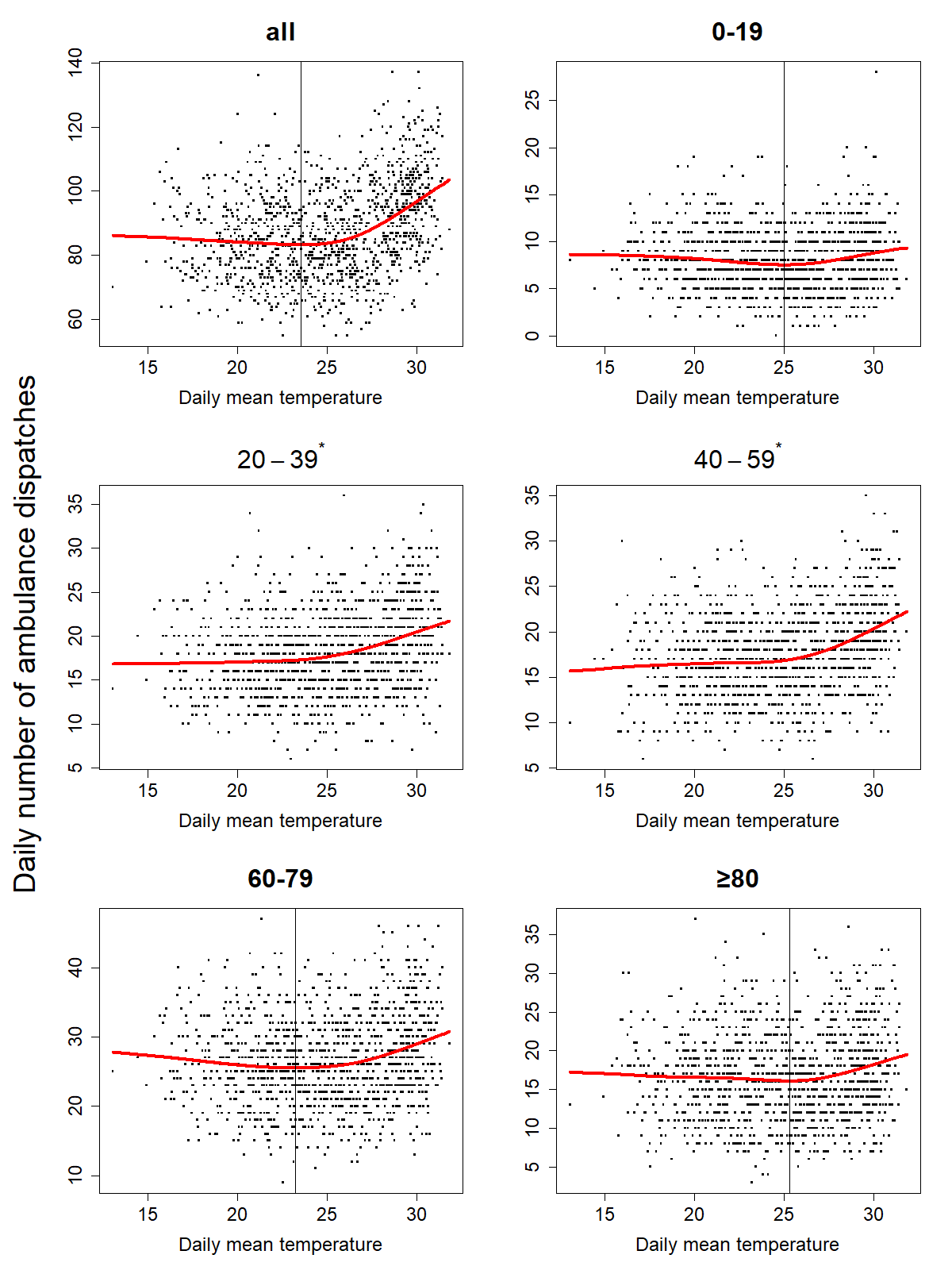


Figure S-1. Daily number of ambulance dispatches and daily mean temperature (°C) with a smoothing line applied by a cubic spline with 4 degrees of freedom for all ages and each age category. The vertical line indicates the optimum temperature.

*Spline curves of 20–39 years and 40–59 years were not U-shaped and did not have an optimum temperature.


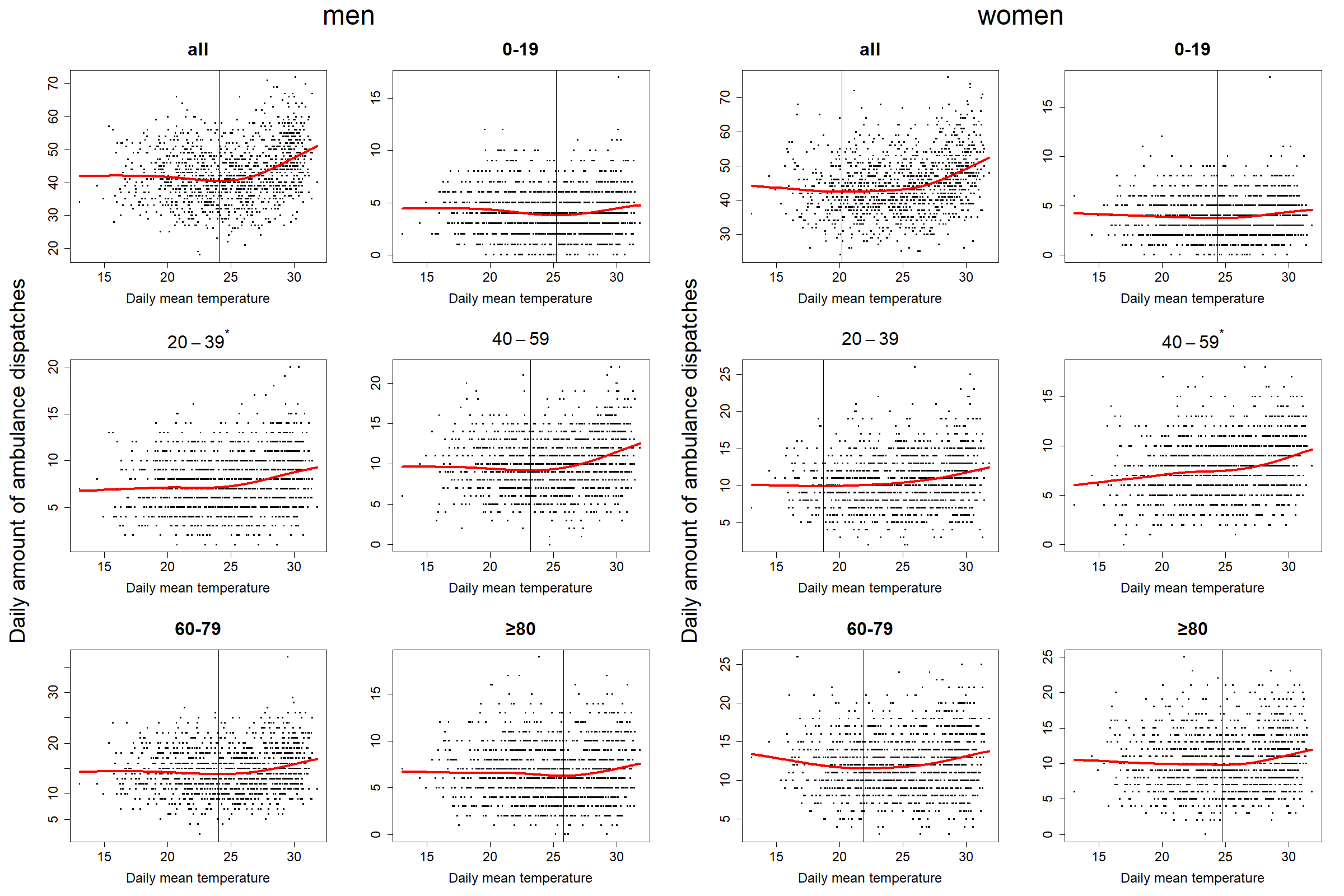


Figure S-2. Daily amount of ambulance dispatches and daily mean temperature (°C) with a smoothing line applied by a cubic spline with 4 degrees of freedom for all ages and each sex and age category. The vertical line indicates the optimum temperature.

*Spline curves of 40–59 years in men and 20–39 years in women were not U-shaped and did not have an optimum temperature.


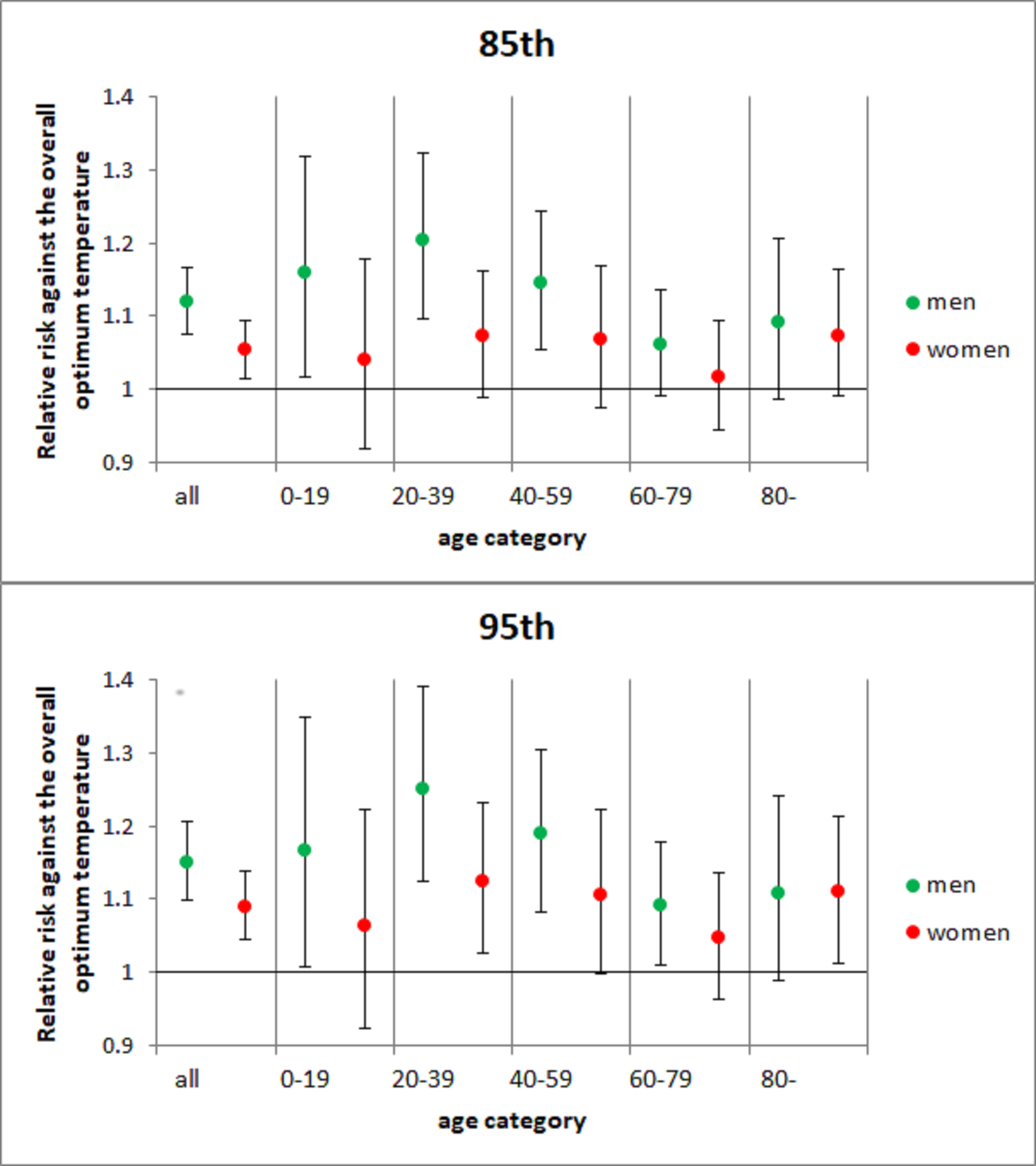


Figure S-3. Relative risks of ambulance dispatches for each sex age category at the 85^th^ (above) and 95^th^ (below) percentile relative to the reference temperature (23.55 ºC, optimum temperature on all ages and all people).
