# Supplementary material for: Effects of high ambient temperature on ambulance dispatches in different age groups in Fukuoka, Japan
Source: Glob Health Action. 2018 Feb 23;11(1):1437882. doi: 10.1080/16549716.2018.1437882 (PMC5827789; doi:10.1080/16549716.2018.1437882)
Supplement: Supplementary material [file ZGHA_A_1437882_SM7165.zip › 180126_kotani_cle_SuppTable.docx]

Table S-1. Distribution of medical conditions requiring ambulance dispatch by age groups.

| Age group | Medical condition of the patients (%) | | | | |
| --- | --- | --- | --- | --- | --- |
|  | Mild | Moderate | Serious | Dead | Others |
| **All** | **42.8** | **51.1** | **6.0** | **0.1** | **0.0** |
| 0–19 | 63.8 | 35.3 | 0.9 | 0.0 | 0.0 |
| 20–39 | 59.3 | 39.6 | 1.1 | 0.0 | 0.0 |
| 40–59 | 46.6 | 47.9 | 5.4 | 0.1 | 0.0 |
| 60–79 | 34.7 | 56.7 | 8.5 | 0.1 | 0.0 |
| ≥80 | 23.7 | 65.7 | 10.3 | 0.3 | 0.0 |

Table S-2. The optimum temperature and its percentile for each sex and age group.

| Sex and age group | Optimum temperature (ºC) | Percentile of optimum temperature |
| --- | --- | --- |
| **Men** | **24.1** | **41** |
| 0–19 | 25.2 | 50 |
| 20–39 | - * | - * |
| 40–59 | 23.2 | 35 |
| 60–79 | 24.0 | 41 |
| ≥80 | 25.8 | 54 |
| **Women** | **20.2** | **13** |
| 0–19 | 24.4 | 43 |
| 20–39 | 18.7 | 7 |
| 40–59 | - * | - * |
| 60–79 | 21.9 | 25 |
| ≥80 | 24.7 | 46 |
| * The pattern was not U-shaped and the optimum temperature was not identified. | | |
